# A Straw Shows Which Way the Wind Blows: A Successful Cannulation of Abnormal Duodenal Papilla

**DOI:** 10.5152/tjg.2026.25493

**Published:** 2026-01-05

**Authors:** Xuanhua Chen, Huanlu Xu, Shenghui Chen, Weigang Gu, Zhijie Wang, Xiaofeng Zhang

**Affiliations:** 1 Department of Gastroenterology, Affiliated Hangzhou First People’s Hospital, School of Medicine, Westlake University, Zhejiang, China; 2 The Fourth School of Clinical Medicine, Zhengjiang Chinese Medical University, Zhejiang, China; 3 Key Laboratory of Integrated Traditional Chinese and Western Medicine for Biliary and Pancreatic Diseases of Zhejiang Province, Zhejiang, China; 4 Hangzhou Institute of Digestive Diseases, Zhejiang, China

## Case Presentation

**Figure 1.**
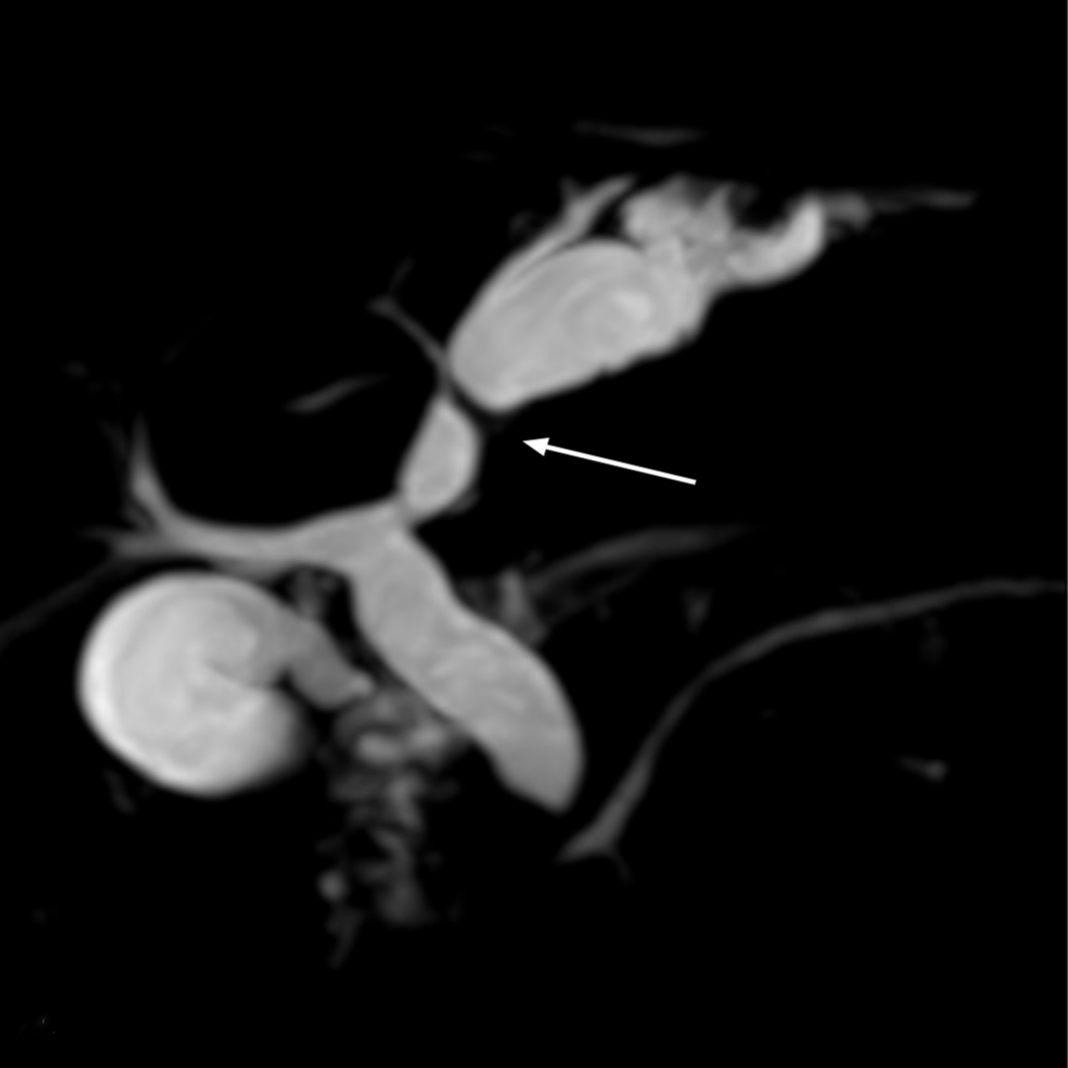
MRCP showing the dilation of the left intrahepatic bile duct and common bile duct (CBD). A localized filling defect (white arrow) can be seen at the proximal left intrahepatic bile duct, dividing the bile duct into two parts.

**Figure 2.**
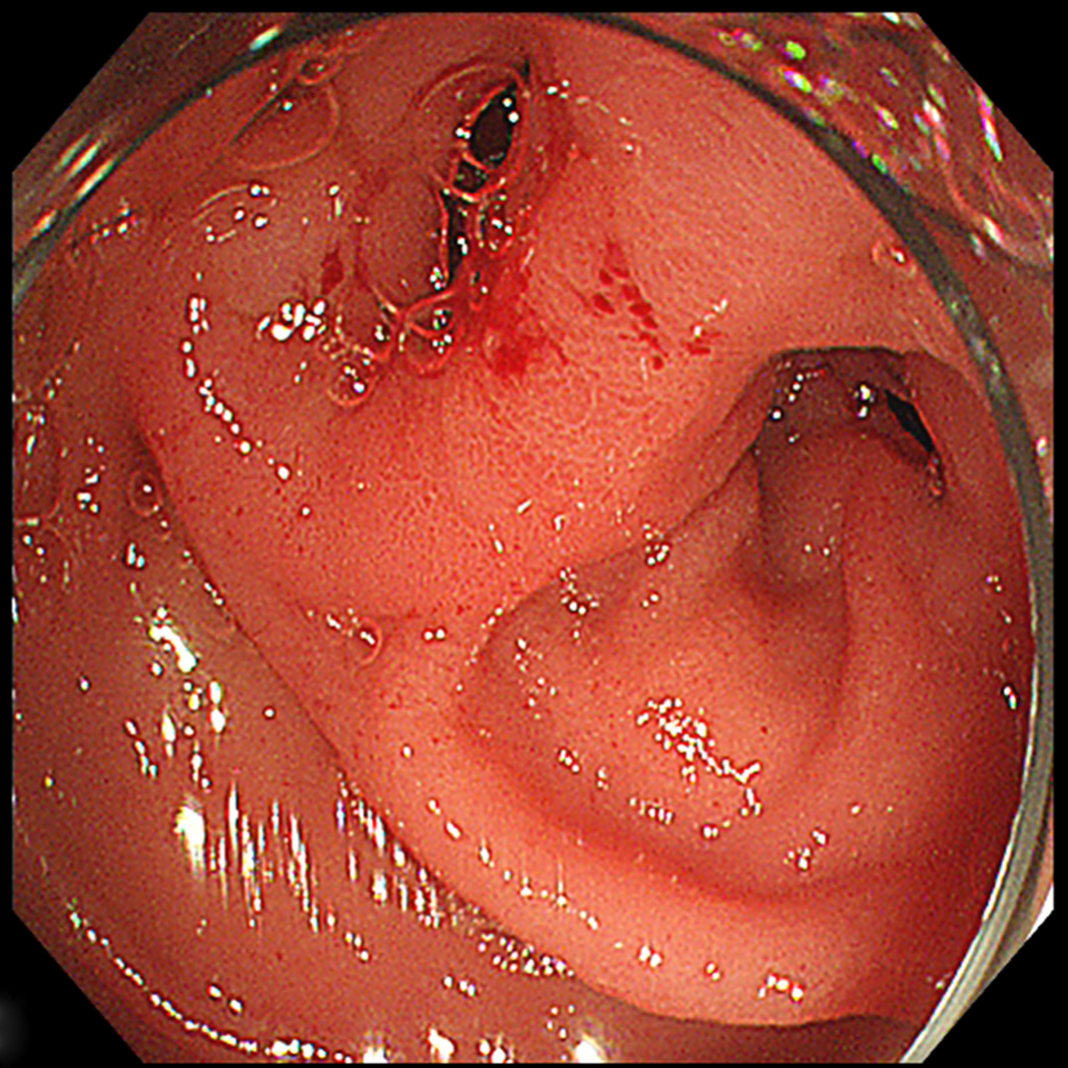
Two entrances of duodenal lumen were found in the descending part of duodenum.

A 56-year-old man was referred to our hospital with a sudden onset of sharp epigastric abdominal pain 3 days ago, which was relieved spontaneously approximately 30 hours later. The patient suffered a similar symptom several years ago and was admitted for acute cholangitis. There were no significant findings in the physical examination and laboratory tests except for dilation of the left intrahepatic bile duct and common bile duct (CBD) identified by magnetic resonance cholangiopancreatography (MRCP). The tumor was suspected to be responsible for the condition, as the lower segment of the CBD was observed to be interrupted (Figure 1). To clarify the diagnosis, endoscopic retrograde cholangiopancreatography (ERCP) was performed. Unexpectedly, tubular duodenal duplication^1^ with 2 entrances of duodenal lumen were found in the descending part (Figure 2). Written informed consent was obtained from the patient for publication of these images and a video.

## Technique

**Figure 3.**
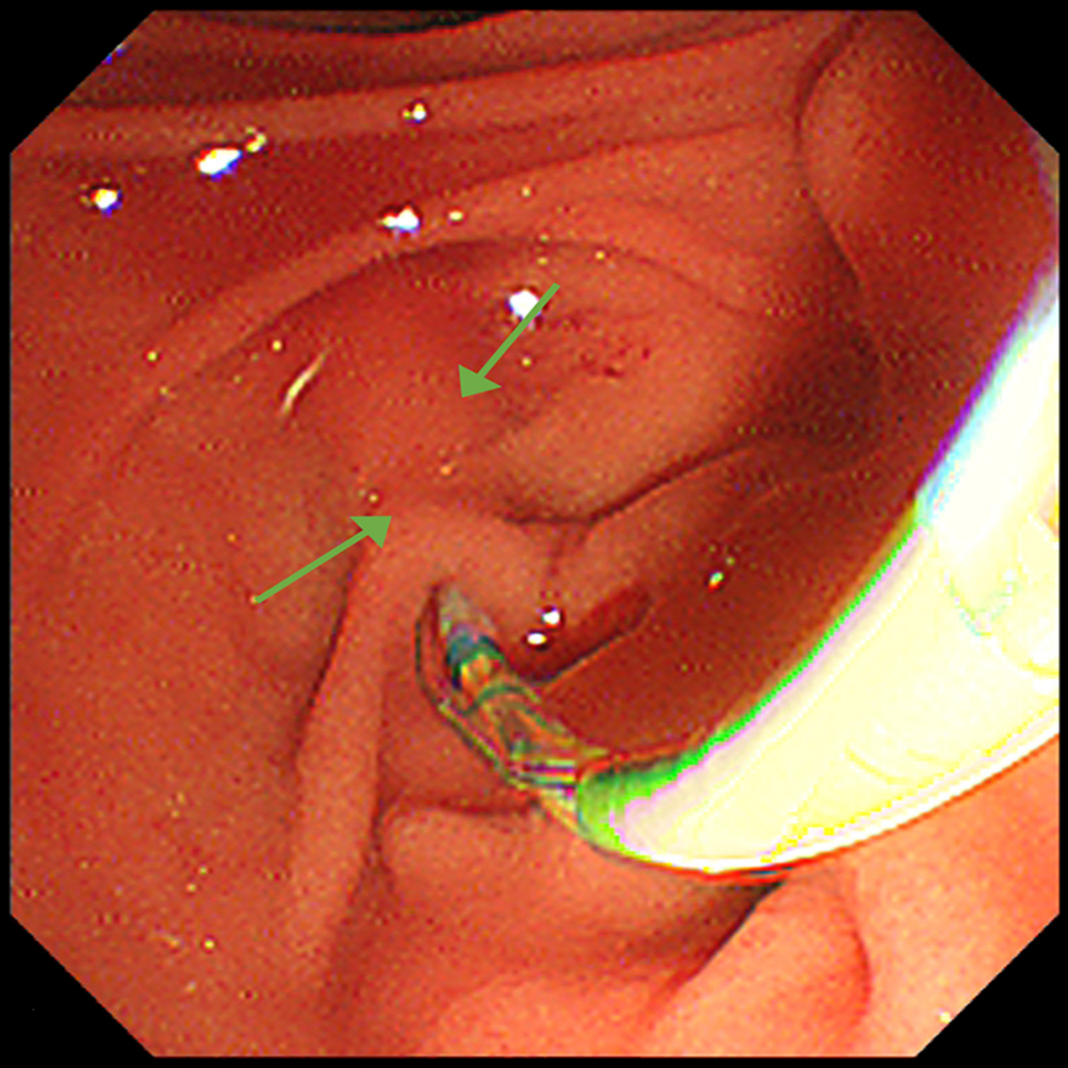
Common bile duct below the intestinal wall was clearly visible.

**Figure 4.**
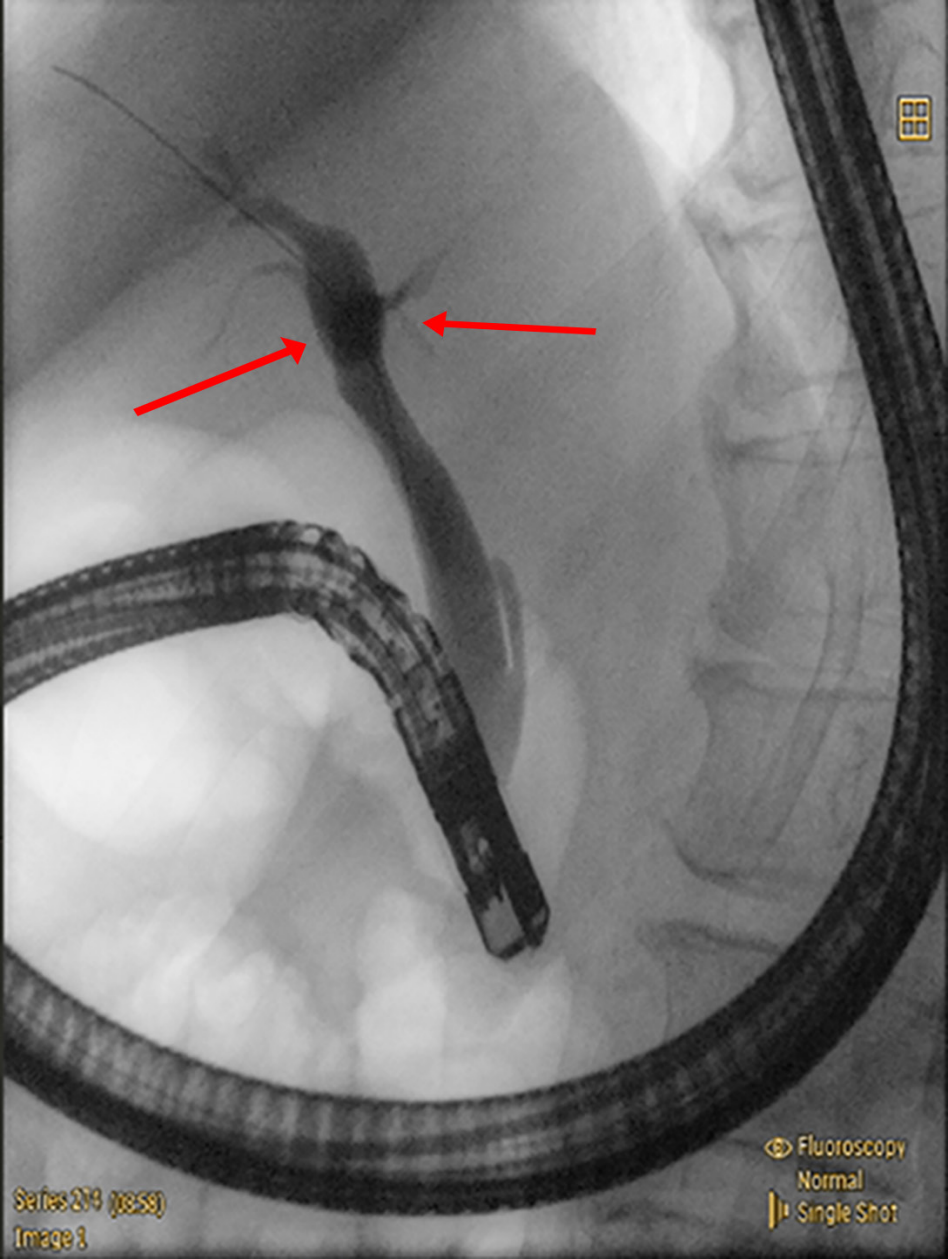
Successful cannulation and cholangiography. Limited by the projection angle, the dilated left intrahepatic bile duct (partially opacified under fluoroscopy, red arrow) and the common bile duct appear overlapped in the image.

Although the endoscope failed to pass through the smaller entrance, it accessed the horizontal segment of the duodenum through the other one. Two entrances were demonstrated to communicate with each other by injecting methylene blue solution from the smaller one. A granular and villous area was observed near the larger entrance and initially considered as the Vater papilla (Supplementary Figure 1). However, bile duct cannulation was unsuccessful despite multiple attempts. Interestingly, the contour of the CBD, which was hidden below the intestinal wall, was clearly visible during a peristaltic contraction of the duodenum (Figure 3). It enabled us to trace the CBD orifice, which is located near the smaller entrance, and successful cannulation. No tumor or stone was found in the following cholangiography (Figure 4). However, there was a proximal stenosis in the left intrahepatic bile duct, which prevented the contrast agent from filling the distal bile ducts, and repeated attempts to traverse it using a guidewire were unsuccessful. Obstruction of the smaller entrance (probably blocked by food debris) and/or the acute-angled connection between the CBD and duodenum was considered responsible for cholangiectasis and abdominal pain by impeding the CBD drainage. Endoscopic sphincterotomy was performed to prevent symptom relapse.

## Conclusion

This is a rare case of tubular duodenal duplication, presenting with obstruction of the lower segment of the CBD and abdominal pain, which was initially suspected as a malignant tumor around the duodenal papilla. Postoperatively, detailed medical history was obtained that the patient had repeatedly experienced upper abdominal discomfort since childhood, which was long managed as “chronic gastritis.” The patient’s previous imaging studies had already demonstrated dilation of the left intrahepatic bile duct and atrophy of the left hepatic lobe (Supplementary Figure 2). We speculate that the congenital anomaly caused chronic partial obstruction of the bile duct, leading to recurrent infections and compensatory dilation. Compared to the right side, the left intrahepatic bile duct is longer and narrower, with a more acute insertion angle into the common hepatic duct. This anatomical configuration may render it more susceptible to bile stasis and infection in the setting of impaired drainage, which could ultimately lead to dilation of the left intrahepatic bile duct and subsequent lobar atrophy. Given that the long-standing chronic inflammation in the left liver lobe may increase the patient’s risk of neoplasia, surgical intervention was recommended; however, the patient declined. The patient is asymptomatic with a follow-up of 2 months after discharge. At last, abnormal anatomy usually increases the difficulty of ERCP procedure. Observation, reasoning, and sometimes luck, are all key to the successful solutions.

## Video 1:

A successful cannulation in a rare case of symptomatic tubular duodenal duplication with abnormal anatomy of duodenum lumen.

## Supplementary Materials

Supplementary Material

## References

[1] Cheng CL Liu NJ Yu MC. Intraluminal tubular duodenal duplication with bleeding. Clin Gastroenterol Hepatol. 2014;12(2):e10 e11. (doi: 10.1016/j.cgh.2013.08.007) 23954648

