# Supplementary material for: A Straw Shows Which Way the Wind Blows: A Successful Cannulation of Abnormal Duodenal Papilla
Source: Turk J Gastroenterol. 2026 Jan 5;37(3):406–8. doi: 10.5152/tjg.2026.25493 (PMC12994422; doi:10.5152/tjg.2026.25493)
Supplement: Supplementary Material [file supplementary_material.pdf]

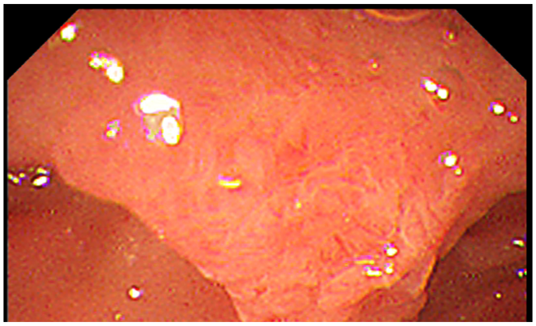

Supplementary Figure 1. Suspected Vater papilla was observed near the larger entrance.

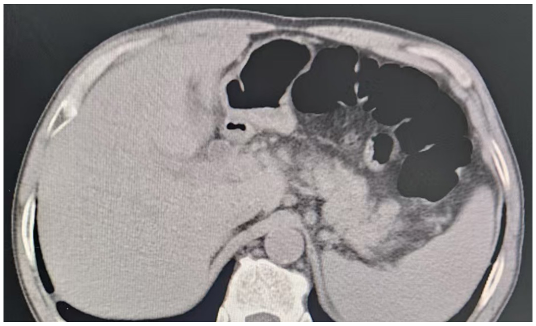

Supplementary Figure 2. Previous CT (six years ago) showed the dilation of the left intrahepatic bile duct and common bile duct, combined with left hepatic lobe atrophy.
